# Validation of a non-food or water motivated effort-based foraging task as a measure of motivational state in male mice

**DOI:** 10.1038/s41386-024-01899-y

**Published:** 2024-06-19

**Authors:** Foteini Xeni, Caterina Marangoni, Megan G. Jackson

**Affiliations:** School of Physiology, Pharmacology and Neuroscience, Biomedical Sciences Building, University of Bristol, BS8 1TD Bristol, UK

**Keywords:** Motivation, Emotion

## Abstract

Disorders of motivation such as apathy syndrome are highly prevalent across neurological disorders but do not yet have an agreed treatment approach. The use of translational behavioural models can provide a route through which to meaningfully screen novel drug targets. Methods that utilise food deprivation in contrived environments may lack the sensitivity to detect deficits in self-initiated behaviour, and may have limited translation to normal behaviour. Animals monitored in more naturalistic environments may display more ethologically-relevant behaviours of greater translational value. Here, we aimed to validate a novel, non-food or water motivated effort-based foraging task as a measure of motivational state in mice. In this task, the mouse can freely choose to exert effort to forage nesting material and shuttle it back to a safe and enclosed environment. The amount of nesting material foraged is used as a readout of motivational state. Acute dopaminergic modulation with haloperidol, amphetamine and methylphenidate, and two phenotypic models known to induce motivational deficits (healthy ageing and chronic administration of corticosterone) were used to validate this task. Consistent with other effort-based decision-making tasks we find that foraging behaviour is sensitive to acute modulation of dopaminergic transmission. We find that both phenotypic models induce differing deficits in various aspects of foraging behaviour suggesting that the task may be used to parse different behavioural profiles from distinct disease phenotypes. Thus, without requiring extended training periods or physiological deprivation, this task may represent a refined and translational preclinical measure of motivation.

## Introduction

Apathy syndrome is a psychiatric syndrome characterised by a reduction in self-initiated/goal-directed behaviour and emotional blunting [1, 2]. It is pervasive across numerous neurological and neurodegenerative diseases, yet does not currently have an agreed treatment approach [3, 4]. This may be in part be due to a lack of understanding of its underlying neurobiology, and a preclinical pipeline through which to screen novel drug targets.

Our currently available preclinical methods for assessing goal-directed behaviour derive from operant conditioning paradigms such as the effort for reward task (EfR) [5]. Here, the rodent is food restricted, and must make an effortful number of operant responses (lever press or nose poke) to receive a palatable food reward, or choose to consume a lower value freely available lab chow. The EfR task has been valuable in advancing our understanding of the neural substrates underlying goal-directed behaviour, demonstrating a key role for the mesolimbic dopaminergic (DA) system [5–8]. However, such operant tasks take weeks-months to train, are run in a contrived environment, with sometimes high levels of food restriction. The purpose of this is to induce a robust and well-defined behavioural response, but may come at a cost to behavioural interpretation. For example, it is unclear whether a deficit in intrinsic self-drive characteristic of apathy can be detected under the pressure of a significant external motivator induced by food restriction, or whether such conditions allow for ‘normal’ behaviour to be observed. Food restriction has also been shown to fundamentally change physiology and brain function, which may impinge on our neural circuits of interest in an unknown way [9]. There is a growing push towards the use of ethological, evolutionarily-conserved behaviours as a focal point of behavioural tasks [10]. By utilising spontaneous, intrinsically rewarding behaviours, we can potentially obtain robust, reliable responses in more complex environments without the need to externally modulate motivational state via food restriction. In this way, motivational state can be assessed in a potentially more translatable context while refining rodent welfare.

In this work, we present a novel, non-food or water motivated behavioural task which draws on the mouse’s intrinsic drive to forage for nesting material. The amount of material foraged is used as a readout of motivational state. Through acute modulation of the dopaminergic system, and the use of phenotypic models previously shown to induce a deficit in reward motivation (healthy ageing and chronic corticosterone (CORT) administration [11, 12]) we aimed to validate the effort-based foraging (EBF) task as a refined measure of motivational state in mice. The D2 receptor antagonist haloperidol was selected to reduce DA transmission, while the psychostimulant amphetamine and dopamine transporter inhibitor methylphenidate were selected to elevate DA transmission. These compounds have been used to validate previous effort based decision making studies [5, 8].

## Methods

### Subjects

8 cohorts of male C57BL/6JOlaHsd mice (Envigo (UK)) and 1 cohort of female C57BL/6JOlaHsd mice were used in these experiments (**see** S1**for summary**). Male mice were singly housed in enriched open-top Techniplast 1284 conventional cages to avoid significant levels of in-fighting commonly observed with this strain and sex and thus minimise any impacts of aggression and associated psychosocial stress. Singly housing male mice may align more closely with the ethology of the species, where individual territories are preferred in more naturalistic environments [13]. Mice underwent twice daily health checks to ensure good welfare throughout experimental testing. Each cage was enriched with a small red house, a wooden chew, a tube, a tube hung from the ceiling and paper shavings (IPS Product Supplies Ltd). Standard laboratory chow (Purina, UK) and water were provided *ad libitum* throughout experiments. Mice were housed in a 12:12 reverse light schedule. All behavioural testing took place in the animal’s active phase (ZT (Zeit Time) 13–21). Sample sizes were based on detecting a large effect size with mean difference and variance based on previous behavioural studies using a similar task and acute pharmacological manipulations and phenotypic models [8, 12]. In all pharmacological studies the experimenter was blind to treatment. In the case of acute pharmacological studies and studies relating to changes in the external environment mice were randomly assigned a treatment group using a within-subject counterbalanced Latin square design. The experimental unit for all studies was the individual animal. All experiments were performed in accordance with Animals Scientific Procedures Act (ASPA, UK) 1986 and were approved by the University of Bristol Animal Welfare and Ethical Review Body. This study used males only to validate the task and greater translatability would be achieved with the use of both sexes. However, to provide some initial insight into translatability across sexes, female task performance was compared to males in a single experiment. C57bl/6 mice were selected due to their widespread use across the fields of neuroscience and pharmacology. It should be noted that there is the potential for variability in foraging activity baseline between mouse strains.

### Effort-based foraging task arena

**Fig. 1:**
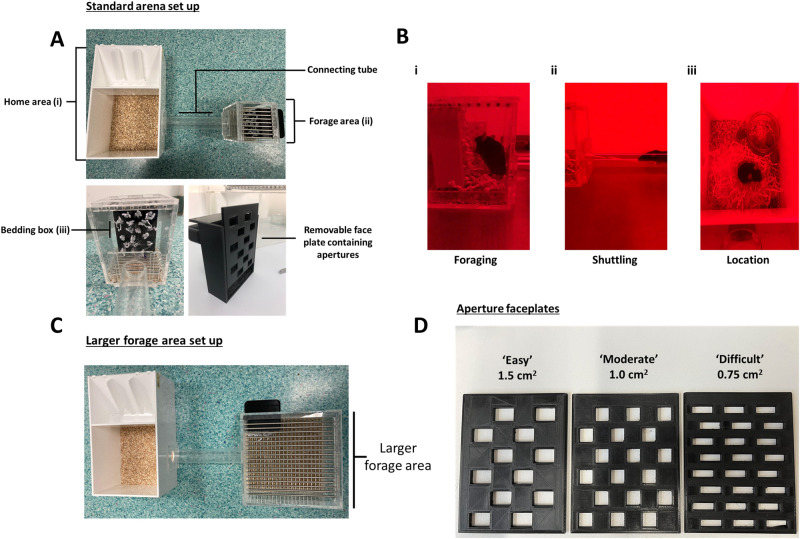
Effort-based forage arena set up. The standard arena **A** consists of four main components. The home area **Ai**, the connecting tube, the forage area **Aii** and the nesting box **Aiii**. The home area contains woodchip on a flat surface, and free access to food and water. It is covered by a detachable lid when in use. The nesting box is filled with 18 g of Sizzlenest. **B** Throughout the task period, the mouse has the option to traverse the tube, forage nesting material from the nesting box by pulling it with their mouth/paws **Bi**, and shuttle it back through the tube **Bii** to the home area **Biii**. **C** In an amended arena set up, a larger forage area is used to assess the impact of stress induced by aversion to open spaces on foraging behaviour (affective reactivity). **D** To modulate effort required to forage nesting material during ‘effort curve’ testing, three different face plates containing different size apertures are used. The moderate aperture is used consistently for acute pharmacological testing.

A foraging arena was developed in house by MGJ to assess forage-based motivation in mice. The apparatus is designed to encourage the mouse to exert effort to forage nesting material and shuttle it back to a safe and enclosed environment. A ‘nesting box’ was developed to provide mice with access to nesting material which requires effort to obtain via varying sizes of apertures. Custom components are available for print here https://github.com/meganjackson13/Bedding-box-3D-files. The standard arena set up is outlined in Fig. 1A. Amended versions of the task for affective reactivity testing and effort curve testing are outlined in Fig. 1C, D. Further arena dimensions and construction details are provided in Supplementary S2, 3.

### Habituation to the effort-based foraging task

Mice were placed in the home compartment of the forage arena containing woodchip only and were left to explore for a period of 10 min. On the second and third day, they were left for a period of 5 min. Woodchip was changed between mice. On the fourth day, they were placed in the forage arena for 4 h. This time, the arena contained a bowl of standard lab chow and their water bottle placed in the main compartment, and a nesting box placed in the foraging area. The nesting box was filled with 18 g of white Sizzlenest (Datesand) (consistent across all studies), which could be pulled through 1.5 cm^2^ apertures. Across all studies the main output measures were total nesting material shuttled to the home area (g) (amount of nesting material pulled (foraged) from the nesting box and taken through the tube to the home area) and percentage nesting material foraged taken to the home area ((total foraged – amount left on the floor of the forage area)/total foraged)*100. Additional detail regarding selection of nesting material is provided in Supplementary S4. The nesting material box is filled with a set amount of Sizzlenest (18 g) and is weighed before the session. After the session is finished the box is weighed again. The amount of nesting material that is pulled from the box but left on top of the barred floor of the forage arena is also weighed. From this we get a readout of total amount of nesting material pulled from the box, and how much has been pulled from the box but not shuttled to the home area.

### Acute pharmacology

16 mice aged 12–14 weeks were used for the haloperidol and i.p amphetamine experiments, and 16 mice aged 13–15 weeks were used for the methylphenidate and oral amphetamine studies. Following habituation mice underwent three 2 h sessions of foraging using the nesting box face plate with 1 cm^2^ apertures which produced a moderate level of difficulty. Sessions were repeated to ensure stability in performance before starting drug testing. An acute study consisted of four 2-h test sessions (vehicle and 3 doses). Each test session was separated by at least 2 days to ensure drug washout between sessions (Supplementary S5). Drugs were administered in a 10 ml/kg dose volume. Haloperidol (0.01–0.1 mg/kg, i.p, pre-treatment time (PT) −60 min, Sigma UK), amphetamine (0.1–0.3 mg/kg, oral and i.p, PT −15 min, Sigma UK) and methylphenidate (1, 3 and 10 mg/kg, oral, −15 min PT, Sigma UK) were used. Drug vehicles are reported in Supplementary S6. Doses and pre-treatment times were selected based on available pharmacokinetic data and previously used doses [8, 14, 15]. Mice were restrained and scruffed for i.p injection using a refined protocol which avoids tail handling outlined in [16]. In the case of oral dosing, drugs were dissolved in 20% condensed milk, and mice were trained to drink voluntarily from a syringe (outlined in Supplementary S7). Changes in locomotor activity following treatment with haloperidol and amphetamine were assessed using a passive infra-red sensor system (further details here Supplementary S8).

### Phenotypic models

#### Chronic corticosterone

In two cohorts of mice run consecutively, half were treated with CORT (*n* = 15) (corticosterone, HBC complex (2.5 mg/100 ml), Sigma Aldrich, UK) in drinking water for 3 weeks prior testing and half were given standard tap water (*n* = 16). Mice on average drank ~ 5 ml water/day and weighed on average 35 g. Following standard habituation including an initial foraging session (1.5 cm^2^ aperture), mice underwent three 2 h foraging sessions under different effort contingencies governed by the size of the nesting box aperture, allowing the formation of an ‘effort curve’. The effort required for foraging was changed by swapping the face plate on the nesting box (Fig. 1D). Mice also underwent two 2 h foraging sessions using the 1.5 cm^2^ aperture where the nesting box was placed in either the enlarged or standard forage area (Fig. 1C). This was to test affective reactivity driven by mice finding more open, novel spaces aversive [17].

#### Healthy ageing

Following standard habituation, 12 young (13–23 weeks old) and 11 aged mice (44–54 weeks old) underwent the effort curve and affective reactivity testing as described above. To account for potential age-related changes in motoric ability, the task was extended to 4 h.

### Males versus females

Females were housed in pairs under housing conditions described above. Following standard habituation, 8 female mice (7 weeks old) and 8 male mice (7 weeks old) underwent effort curve testing as described above.

### Environmental changes

#### Free nesting material

16 mice (35 weeks old) underwent two 2 h forage sessions using the 1 cm^2^ (mid-size) aperture with either the presence or absence of 10 g of freely available Sizzle nesting material, placed in the main compartment.

#### Warming

A heat mat (NEKOSUKI) was placed under the main compartment of the forage arena, and the floor was warmed to a temperature of ~29 °C, the threshold for the thermoneutral zone for mice [18]. 12 mice (30 weeks old) were habituated to the warmed arena in a 20 min session without the nesting box the day preceding the test sessions. The mice underwent two 4 h test sessions in either warmed or standard conditions.

### Statistical analysis

Data were tested for normality using a Shapiro-Wilk test. Where data were normally distributed and required single factor analysis, a repeated measure (RM) one-way ANOVA (Geisser-Greenhouse corrected) was used. Where significant main effects were observed (*p* < 0.05), Dunnett’s post-hoc comparisons were carried out. Where data violated normality, a Friedman test was applied. Where a significant main effect was observed, Dunn’s post hoc comparisons were carried out. Trend level main effects (*p* < 0.1) are reported but not further analysed. Where two factor analysis was required, a RM two-way ANOVA was applied. Where significant main effects occurred, these were tested using Sidak post-hoc comparisons. Where two groups were compared within subject, a paired *t*-test was used. Outliers were defined as data points ± 2SDs away from the mean. Outliers were replaced with the group mean to permit repeated measures analysis. If data was missing due to random errors in recording then a mixed model was applied instead. All instances of outlying/missing data points are provided in Supplementary S9. Data were graphed and analysed using GraphPad Prism v 10.0.03.

## Results

### Foraging behaviour is sensitive to acute dopaminergic modulation

**Fig. 2:**
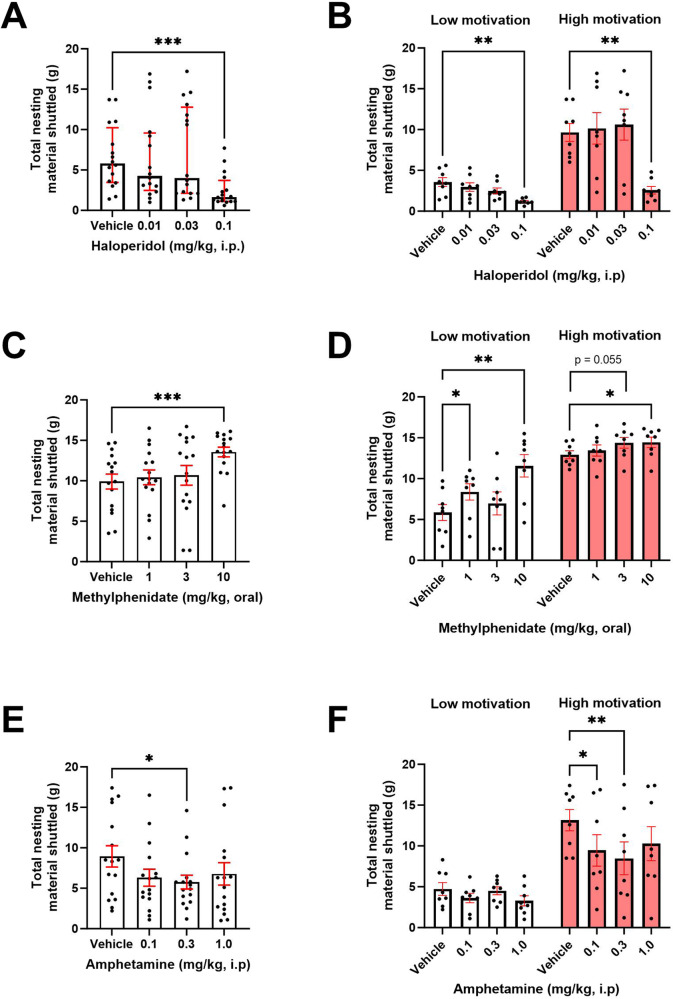
Foraging behaviour is sensitive to acute dopaminergic modulation. Haloperidol reduced total nesting material shuttled **A**. This effect was consistent across low and high performers **B**. Methylphenidate increased total nesting material shuttled **C**. Drug effect was differentially modulated across low/high performers **D**. Amphetamine (i.p) reduced total nesting material shuttled **E**. This effect was specific to high performers **F**. Bars are mean ± SEM or median ± interquartile range where data are non-parametric. *p < 0.05, **p < 0.01, ***p < 0.001.

Haloperidol (0.01–0.1 mg/kg) had a main effect on total nesting material shuttled (X_2_ = 17.15, *p* = 0.0007, where 0.1 mg/kg reduced nesting material shuttled (*p* = 0.0005)) (Fig. 2A). To assess a potential interaction between drug effect and intrinsic motivational state, the cohort was then stratified into ‘high’ and ‘low’ performers using a median split of nesting material shuttled under vehicle conditions. There was a main effect of drug (F_(2.171, 30.40)_ = 11.88, *p* = 0.0001), and a drug*motivational state interaction (F_(3,42)_ = 4.856, *p* = 0.0055). 0.1 mg/kg reduced nesting material shuttled in both groups (*p* = 0.0051 and *p* = 0.0011 respectively) (Fig. 2B). There was no effect of drug on locomotor activity (*p* > 0.05) (Supplementary S10).

Methylphenidate (1–10 mg/kg) had a main effect on total nesting material shuttled (F_(2.362,35.42)_ = 7.353, *p* = 0.0013, where 10 mg/kg increased nesting material shuttled (*p* = 0.0003)) (Fig. 2C). When stratified by motivational state there was a main effect of drug (F_(2.598,36.37)_ = 10.15, *p* = 0.0001), and a drug*motivational state interaction (F_(3,42)_ = 4.992, *p* = 0.0047) on nesting material shuttled. 1 and 10 mg/kg increased nesting material shuttled in the lower motivation group (*p* = 0.0227 and *p* = 0.0044 respectively), while 10 mg/kg increased foraging behaviour (*p* = 0.0161) in the higher group, with a trend-level effect at 3 mg/kg (*p* = 0.0551) (Fig. 2D).

There was a main effect of amphetamine (i.p) (0.1–1 mg/kg) on nesting material shuttled (F_(2.836, 42.54)_ = 3.541, *p* = 0.0243), where 0.3 mg/kg decreased nesting material shuttled (*p* = 0.0122) and 0.1 mg/kg trended towards a decrease (*p* = 0.0607) (Fig. 2E). When stratified by motivational state, there was a main effect of drug on nesting material shuttled (F_(2.061, 28.86)_ = 4.767, *p* = 0.0155) and a drug*motivational state interaction (F_(3,42)_ = 3.065, *p* = 0.0382). These effects were specific to the high performance group, where both 0.1 mg/kg and 0.3 mg/kg decreased nesting material shuttled (*p* = 0.0443 and *p* = 0.0049 respectively) (Fig. 2F). There was no effect of drug on locomotor activity (*p* > 0.05) (Supplementary S9). Oral amphetamine had no effect (Supplementary S11).

Analysis of % of foraged nesting material taken to the home area (hereafter referred to as % nesting material shuttled) revealed no effect of drug (*p* > 0.05) under any drug condition (Supplementary S12).

### Foraging behaviour is sensitive to phenotypic models of motivational deficit

**Fig. 3:**
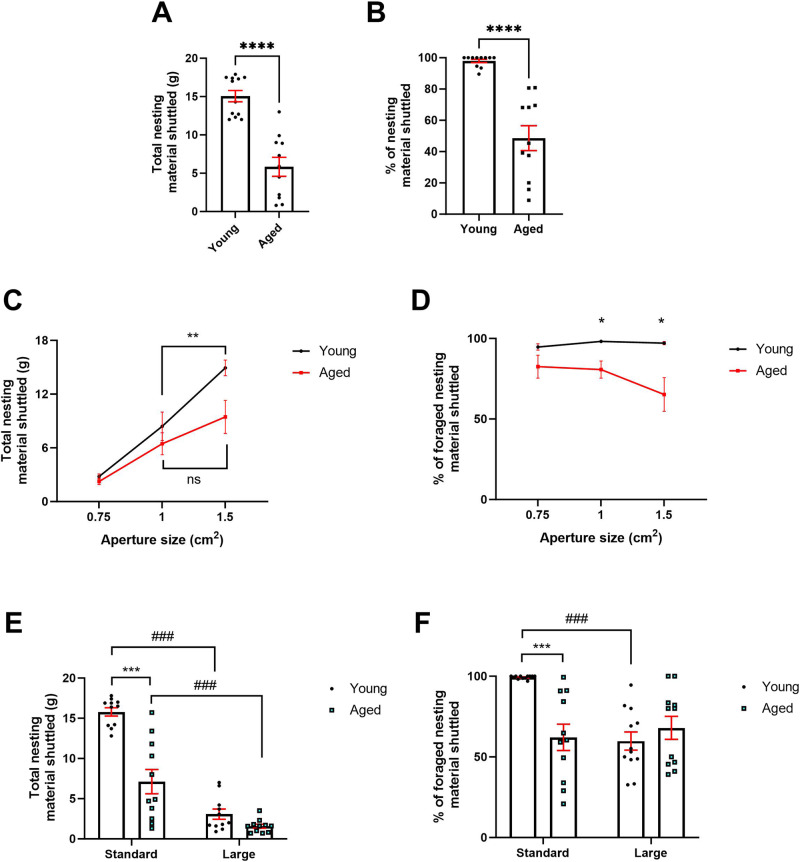
Aged mice show impairments in modulation of foraging behaviour under different effort and affective contingencies. In an initial foraging session, aged mice showed both a reduction in total nesting material shuttled **A** and % nesting material shuttled **B**. Changing aperture size reduced total nesting material shuttled in young mice only **C**. Aged mice showed a reduction in % nesting material shuttled specific to aperture size **D**. A larger forage area reduced nesting material shuttled in both groups. Aged mice foraged less than younger mice in the standard forage area only **E**. A larger forage area reduced % nesting material in younger mice only **F**. Bars are mean ± SEM, **p < 0.01, ***p < 0.001, ###*p* < 0.001 (within-subject comparison).

In an initial foraging session, aged mice shuttled less nesting material than younger mice (t_(21)_ = 6.505, *p* < 0.0001) and showed a reduction in % nesting material shuttled (t_(20)_ = 6.141, *p* < 0.0001) (Fig. 3A, B). Mice then underwent a foraging session under different effort contingencies governed by the size of the nesting box aperture, allowing the formation of an ‘effort curve’. Here, there was a main effect of age (F_(1,20)_ = 4.790, *p* = 0.0407), aperture (F_(1.969,39.39)_ = 47.3, *p* < 0.0001), and an age*aperture interaction (F_(2,40)_ = 3.242, *p* = 0.0496) on total nesting material shuttled. There was a trend towards aged mice foraging less nesting material at the widest aperture only (*p* = 0.0518). Young mice foraged less nesting material from the 1 cm^2^ aperture than the 1.5 cm^2^ (*p* = 0.003) but aged mice did not (*p* > 0.05) (Fig. 3C). There was a main effect of age on % nesting material shuttled (F_(1,20)_ = 9.677, *p* = 0.0055) and an aperture*age interaction (F_(2,40)_ = 3.285, *p* = 0.0478). Aged mice had a lower % nesting material shuttled compared to younger mice at both the 1 cm^2^ aperture and the 1.5 cm^2^ aperture (*p* = 0.0247 and *p* = 0.0361 respectively) (Fig. 3D).

Next, the impact of the size of the foraging area on foraging behaviour was assessed. There was a main effect of forage area size (F_(1,20)_ = 104.8, *p* < 0.0001), age (F_(1,20)_ = 36.40, *p* < 0.0001) and an age*size interaction (F_(1,20)_ = 16.15, *p* = 0.0007) on total nesting material shuttled. Aged mice shuttled less nesting material than younger mice (*p* < 0.0001) under standard conditions only. Both groups foraged less in the larger versus standard forage area (*p* < 0.0001 and *p* = 0.0006 respectively) (Fig. 3E). There was a main effect of age (F_(1,21)_ = 4.416, *p* = 0.0478), size (F_(1,21)_ = 12.20, *p* = 0.0022) and a size*age interaction (F_(1,21)_ = 22.19, *p* = 0.0001) on % nesting material shuttled. In the standard forage area only, aged mice showed a lower % nesting material shuttled (*p* = 0.0001) compared to younger mice. % nesting material shuttled was reduced in the larger area versus the standard area in young mice only (p < 0.0001) (Fig. 3F).

**Fig. 4:**
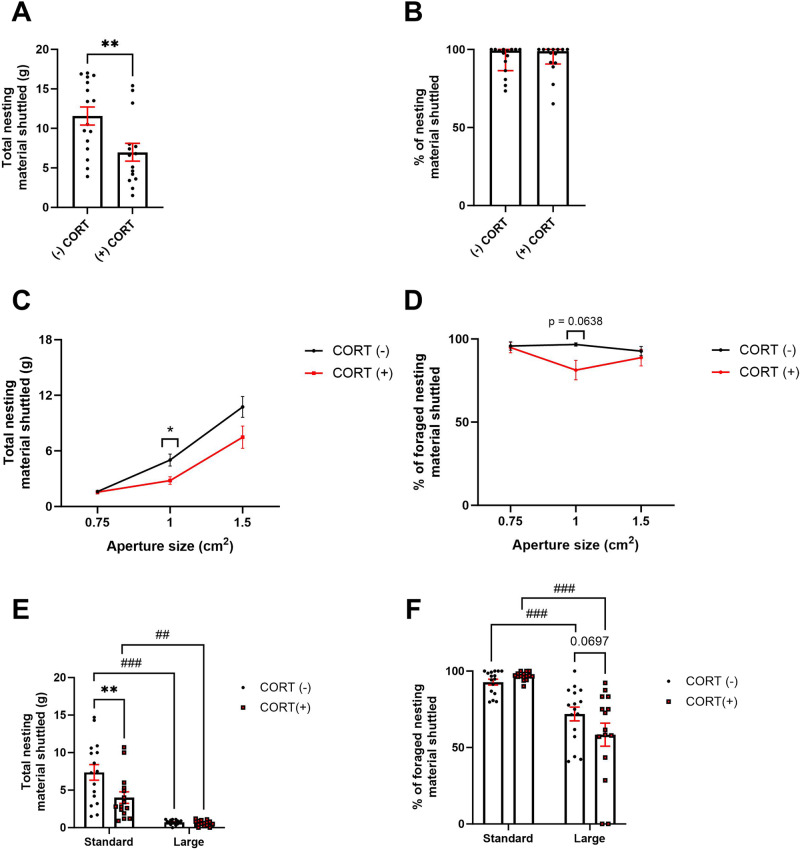
CORT-treated mice show impairments in modulation of foraging behaviour under different effort and affective contingencies. In an initial foraging session, treated mice showed a reduction in total nesting material shuttled **A** but not % nesting material shuttled **B**. Reducing aperture size reduced total nesting material shuttled in both groups, and treated mice shuttled less than non-treated mice at the 1 cm^2^ aperture **C**. There was no difference in % nesting material shuttled at different apertures **D**. Both groups showed reduced total nesting material shuttled in a larger forage area. Group difference disappeared using the larger forage area **E**. % nesting material shuttled was reduced in the larger forage area in both groups **F**. Bars are mean ± SEM, **p < 0.01, ***p < 0.001, ##*p* < 0.01, ###*p* < 0.001 (within-subject comparison).

In an initial session, CORT treated mice shuttled less nesting material than non-treated mice (t_(29)_ = 2.853, *p* = 0.0079) (Fig. 4A) but there was no difference in % nesting material shuttled (*p* > 0.05) (Fig. 4B). When assessed using the effort curve there was a main effect of aperture (F_(1.352, 37.85)_ = 72.83, *p* < 0.0001), treatment (F_(1,28)_ = 5.981, *p* = 0.021) and an aperture*treatment interaction (F_(2,56)_ = 3.274, *p* = 0.0452) on nesting material shuttled. Treatment reduced total nesting material shuttled at the 1 cm^2^ aperture only (*p* = 0.0234) (Fig. 4C). There was a main effect of aperture (F_(1.86,53.02)_ = 3.515, *p* = 0.040) and an aperture*treatment interaction (F_(2,57)_ = 5.534, *p* = 0.0064) on % nesting material shuttled. There was a trend towards a reduction in the treated group at 1 cm^2^ only (*p* = 0.0638). In the treated group only there was a reduction at 1 cm^2^ compared to both 0.75 cm^2^ and 1.5 cm^2^ (*p* = 0.015 and *p* = 0.0345 respectively) (Fig. 4D).

Changing forage area size revealed a main effect of size (F_(1,29)_ = 63.70, *p* < 0.0001), treatment (F_(1,29)_ = 6.399, *p* = 0.0171) and a size*treatment interaction (F_(1,29)_ = 6.619, *p* = 0.0155). Treatment reduced total nesting material shuttled compared to non-treated mice in the standard area only (*p* = 0.0013). Both groups foraged less in the larger forage area compared to the standard (*p* < 0.0001 and *p* = 0.0015 respectively) (Fig. 4E). There was a main effect of size (F_(1,29)_ = 50.34, *p* < 0.0001) and a size*treatment interaction (F_(1,29)_ = 4.409, *p* = 0.0446) on % nesting material shuttled. There was a trend-level reduction in % nesting material shuttled in treated mice versus non-treated mice in the large forage area only (*p* = 0.0697). The larger forage area reduced % nesting material shuttled in both treated and non-treated groups (*p* < 0.0001 and *p* = 0.0024 respectively) (Fig. 4F).

**Fig. 5:**
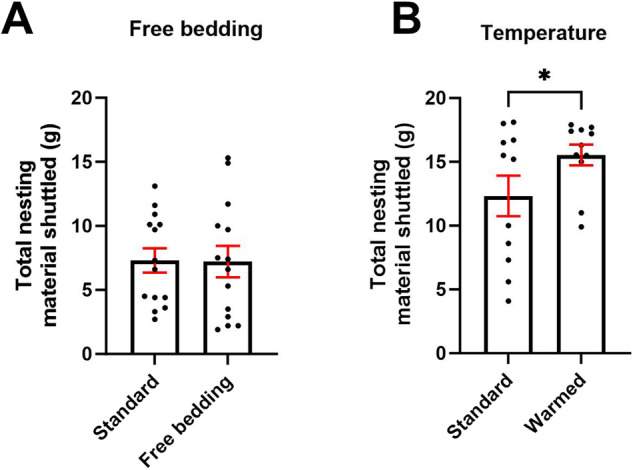
Modulation of aspects of the forage environment change foraging behaviour. There was no effect of freely available nesting material on total nesting material shuttled **A**. Warming the environment increased nesting material shuttled **B**. Bars are mean ± SEM, *p < 0.05.

To investigate the driving factors of foraging behaviour, aspects of the forage environment were modulated. Providing 10 g of freely available Sizzle nesting material had no effect on total shuttled nesting material (*p* > 0.05) (Fig. 5A). Warming the arena increased nesting material shuttled (t_(10)_ = 2.398, *p* = 0.0375) (Fig. 5B).

To investigate the effect of sex on foraging behaviour, males and females underwent testing in the effort curve paradigm. Females foraged and shuttled less nesting material than males at the smallest and largest apertures but not the moderate aperture utilised in the pharmacology manipulations (Supplementary S13A, B). Females showed a reduced % of foraged nesting material shuttled compared to males at all aperture sizes (Supplementary S13C).

## Discussion

Haloperidol and amphetamine (i.p) decreased nesting material shuttled, while methylphenidate increased nesting material shuttled. Oral administration of amphetamine had no effect. Healthy ageing reduced nesting material shuttled, blunted effort modulation and blunted change in shuttle behaviour in a more aversive environment. Chronic corticosterone similarly reduced nesting material shuttled and showed a particular sensitivity to the effort induced by the moderate difficulty aperture. Devaluation of the nesting material via provision of free nesting material and warming did not reduce foraging. The following discussion will consider how these findings contribute to our understanding of this task as a translational measure of motivated behaviour in mice.

### Foraging behaviour is sensitive to acute dopaminergic modulation

Consistent with other measures of motivational state such as the effort for reward (EfR) task, we show foraging behaviour is sensitive to modulation of DA transmission. Haloperidol has previously been shown to reduce the number of high effort, high value reward trials in the EfR task and reduce effortful responding in the progressive ratio task [5, 8, 15, 19]. At the same dose, we found a reduction in nesting material shuttled to the home box independent of changes to locomotion. Methylphenidate (MPH) has been shown to have a positive effect in the EfR task and effort-based decision making in humans [8]. Consistent with these findings, methylphenidate increased nesting material shuttled. It has previously been shown that forage-related activity in the rat is similarly sensitive to changes in mesolimbic DA transmission, suggesting a potential role of this pathway in foraging behaviour [20].

Treatment with amphetamine (i.p) reduced nesting material shuttled, contrasting findings reported in operant conditioning tasks where an increase in effortful responding was reported [8, 15, 21]. This may be driven by differences in task environment. Low dose amphetamine increases task engagement by stabilising task neural trajectories, making it difficult to shift between different behaviours within-task [22]. These effects are conducive to a contrived environment where behavioural options are limited, but may be impairing in complex environments. Oral amphetamine had no effect on foraging behaviour. It has previously been shown that speed of delivery of DA modulators engage distinct neural circuits [23]. The EBF task may be sensitive to differing neurotransmitter dynamics mediated by drug pharmacokinetics in addition to absolute changes in DA levels.

A defining principle of goal-directed behavioural tasks such as the EfR task is that the motivated behaviour can be diminished by devaluing the reward e.g., by pre-feeding. The motivating aspect of the EBF task may be driven by thermoregulation via nest building. However devaluation of the nesting material via warming the arena, and provision of non-effortful access to nesting material, did not reduce effortful foraging. This suggests the engagement in foraging behaviour itself is intrinsically rewarding. This may be more conducive to disorders of self-initiation such as apathy, and gives greater access to individual variability in intrinsic motivational state. There is the potential for the behaviour to be driven by a comparative lack of environmental complexity in the home cage versus the wild leading to a degree of restriction [24]. However, we provide enriched housing including nesting and bedding material to allow the mice to exert their natural nest building behaviour outside of the task context. This factor could be explored in more detail by investigation of environmental complexity in the home cage on foraging motivation in the task.

Stratification of mice by performance revealed that mice in the lower motivation group were more sensitive to lower doses of MPH compared to higher motivated mice. Similarly, amphetamine (i.p) was impairing only in the higher motivation group. This may be driven by differing basal levels of DA within motivation groups which provide differing thresholds for drug response. The drugs used also modulate the noradrenergic system. Use of specific inhibitors are necessary to parse the role of these systems in motivated behaviour more effectively. These data serve as a springboard for demonstrating that motivational grouping may impact on drug effect. However, determining motivation level using a continuous metric such as a score based on performance in a range of other motivation-based tasks may provide insight into motivational grouping and drug effect on a more granular scale while accounting for limitations associated with the median-split approach.

### Foraging behaviour is sensitive to phenotypic models of reward motivation deficit

Ageing has been shown to induce behaviours relevant to apathy syndrome including a deficit in reward motivation measured by the EfR task [12, 25]. Chronic treatment with corticosterone (CORT) is thought to mimic chronic stress or reflect hypercortisolemia found in patients with major depressive disorder (MDD). It has been shown to induce deficits in reward-related domains and exaggerated emotional reactivity relevant to MDD [26].

Normal ageing and CORT treatment reduced nesting material shuttled in an initial foraging session at the widest aperture consistent with motivational deficit. Operant based methods revealed little to no effect of CORT treatment on motivational state [8, 11], suggesting that the EBF task is sensitive to behavioural changes that may otherwise go undetected. Aged mice did not adjust their foraging behaviour relating to total nesting material shuttled in response to a more effortful aperture, suggesting a blunting in effort-based behavioural modulation. This deficit was not observed in CORT-treated mice. Failure to modulate effortful behaviour has been reported in other patient populations with apathy [27] but not MDD. It is important to note that in some models such as ageing it is difficult to disentangle physical ability from motivation. This is a primary reason for using within-subject modulation of the task environment to assess behaviour in phenotypic models in addition to between-subject comparison to their ‘healthy’ counterparts. In this way, their ‘ability’ to do the task remains consistent, and their response to different requirements can be compared to their own baseline. In addition, by using two different phenotypic models we mitigate the risk of findings driven by confounding factors affecting any one model.

Aged but not CORT-treated mice showed a reduction in the percentage of nesting material foraged shuttled to the home box, suggesting transportation of nesting material may reflect an additional effortful component of foraging behaviour differentially modulated by disease state. Indeed, percentage of nesting material shuttled to the home area was differentially modulated by effort in the aged group suggesting shuttling of nesting material represents an additional effortful component. Greater levels of nesting material are foraged at the moderate and easy apertures and thus require greater volumes to transport.

In addition to a reduction in reward motivation, affective reactivity becomes blunted in apathy syndrome and exaggerated in MDD [28]. Both age groups and CORT-treated mice showed a robust reduction in nesting material shuttled when the nesting box was placed in a larger (and hence more aversive) forage area. Along with a reduction in nesting material foraged, younger mice also showed a reduction in percentage of foraged nesting material shuttled in the large forage area context, suggesting this measure has an additional affective component. Transportation of nesting material may increase time spent in the open (more aversive) environment and therefore represent a greater level of ‘risk’. This measure was unaffected in the aged mice irrespective of the larger environment which may be reflective of affective blunting consistent with an apathy phenotype. It is important to note that aged mice showed a lower percentage of nesting material shuttled in the standard arena compared to younger mice suggesting additional factors may affect baseline behaviour. However, this lack of change across environmental condition is unlikely to be driven by an age-related impairment in ability to shuttle nesting material as we observe this output modulated in other contexts including the effort curve paradigm.

In contrast, CORT treated mice trended towards a greater reduction in percentage of nesting material shuttled potentially suggestive of exaggerated affective reactivity consistent with MDD rather than apathy syndrome [28]. Thus, by changing aspects of the foraging environment we may parse different behavioural profiles relating to motivation and affect from distinct disease phenotypes. While these data reveal potentially dissociable components of foraging and decision-making, understanding what drives these behaviours is a crucial avenue for future work.

We find that females are similarly sensitive to aperture size (and by extension, effort requirement) in terms of total nesting material foraged. There was no difference in performance at the moderate aperture which we utilise in drug studies, suggesting male and female data may be combined in pharmacological studies. Males and females do show striking differences in terms of shuttling behaviour. While males shuttled a majority of foraged nesting material, females chose to leave it in the open. The reason for this requires further exploration but may be driven by sex differences in social behaviour, where females may prefer to build a nest in the open with the cage mate nearby. This provides further evidence that the % of foraged nesting material shuttled measure contains an additional affective component. Overall, this task provides an output measure that is similarly modulated in both sexes but crucially is also sensitive to sex differences in foraging behaviour.

### Future perspectives

We show that total nesting material shuttled is sensitive to phenotypic models and acute drug treatment. However, there is likely to be further insight gained into foraging behaviour within a single test session. The use of a transparent forage area is conducive to video recording of behaviour. The use of this data alongside open source AI software such as Deeplabcut could provide additional output measures including bouts of foraging behaviour across time. This could provide insight into behaviours relating to sustained engagement and behavioural switching.

As described above, while the principle metric of the task is the amount of nest material pulled from the box and shuttled to the home area, we find certain task environments, phenotypes and sex induce a propensity to exert effort to pull nesting material from the box, but leave a proportion of foraged material in the open forage area. While these data suggests this may have an affective component, the reason for this behaviour requires further study. These relatively simple readouts provide an exciting opportunity to explore the granular components of motivation/decision-making using a naturalistic behaviour. These components may be parsed in greater detail in future work by changing aspects of the foraging environment and further pharmacology.

## Conclusion

Using acute dopaminergic modulation along with two phenotypic models of motivational deficit we investigated the utility of the EBF task as a measure of motivational state in mice. We show that in line with other effortful decision making paradigms, the EBF is sensitive to DA manipulation suggesting some involvement of the mesolimbic DA system. Using healthy ageing and chronic corticosterone treatment we demonstrate the EBF task is a sensitive measure of motivational deficit and potential affective changes in relevant phenotypic models previously undetected with other methods. Foraging behaviour was not reduced when the nesting material was devalued, suggesting engaging in foraging itself is intrinsically rewarding. Thus, non-food or water motivated foraging may represent a high throughput and refined method for assessment of motivational state.

## Supplementary information

Supplemental material

## Data Availability

All data used in this manuscript is available via this link https://osf.io/6nykz/.
