# Supplementary material for: Validation of a non-food or water motivated effort-based foraging task as a measure of motivational state in male mice
Source: Neuropsychopharmacology. 2024 Jun 19;49(12):1883–91. doi: 10.1038/s41386-024-01899-y (PMC11479259; doi:10.1038/s41386-024-01899-y)
Supplement: Supplementary file 1 — Supplemental material [file 41386_2024_1899_MOESM1_ESM.docx]

**S1**

| Experiment | Length | Aperture size | Cohort | N number | Age |
| --- | --- | --- | --- | --- | --- |
| Free nesting material | 2h | Mid | MJ16 | 16 | 35 weeks |
| Temp study | 4h | Large | MJ15 | 12 | 30 weeks |
| Age: First session | 4h | Large | MJ15/JD5 | 12/group | 13 weeks (Y) 44 weeks (O) |
| Age: Effort curve | 4h | All | MJ15/JD5 | 12/group | 23 weeks (Y) 54 weeks (O) |
| Age: Big vs std | 4h | Large | MJ15/JD5 | 12/group | 19 weeks (Y) 50 weeks (O) |
| CORT: First session | 4h | Large | MT1/BR1 | 16/group | BR1 35 weeks, MT1 18 weeks |
| CORT: Effort curve | 2h | All | MT1/BR1 | 16/group | BR1 35 weeks, MT1 19 weeks |
| CORT: Big vs std | 2h | Large | MT1/BR1 | 16/group | BR1 37 weeks, MT1 21 weeks |
| M v F: Effort curve | 2h | All | MJ21/22 | 8/group | 7 weeks |
| Haloperidol i.p | 2h | Mid | MJ16 | 16 | 12 weeks |
| Amphetamine i.p | 2h | Mid | MJ16 | 16 | 14 weeks |
| Amphetamine oral | 2h | Mid | MJ17 | 16 | 15 weeks |
| Methylphenidate oral | 2h | Mid | MJ17 | 16 | 13 weeks |

***S1. Summary of n numbers and ages per experiment.***

**S2**

| **Component** | **Dimensions** |
| --- | --- |
| Home area | W 17.5 cm, L 30.0 cm, H 13.0 cm |
| Home area lid | W 18.1 cm, L 18.1 cm, H 18.1 cm |
| Connecting tube | L 21.0 cm |
| Forage area | W 10.0 cm, L 10.0 cm, H 13.0 cm |
| Forage area (large) | W 21.0 cm, L 21.0 cm, H 14.0 cm |
| Nesting material box body (3D printed) | W 3.5 cm, L 8.0 cm, H 10.0 cm |
| Nesting material box face plate (3D printed) | W 0.3 cm, L 7.8 cm, H 10.0 cm |
| Nesting material box lid (3D printed) | W 3.5 cm, L 7.8 cm, H 0.3 cm |
| Magnet bar (3D printed) | W 2.0 cm, L 8.0cm, H 3.0 cm |
| ‘Easy apertures’ (1.5 cm^2^) | W 1.5 cm H 1 cm |
| ‘Moderate apertures’ (1 cm^2^) | W 1 cm H 1 cm |
| ‘Difficult apertures’ (0.75 cm^2^) | W 1.5 cm H 0.5 cm |

***S2. Component dimensions and 3D print files for forage arena.***

**S3**


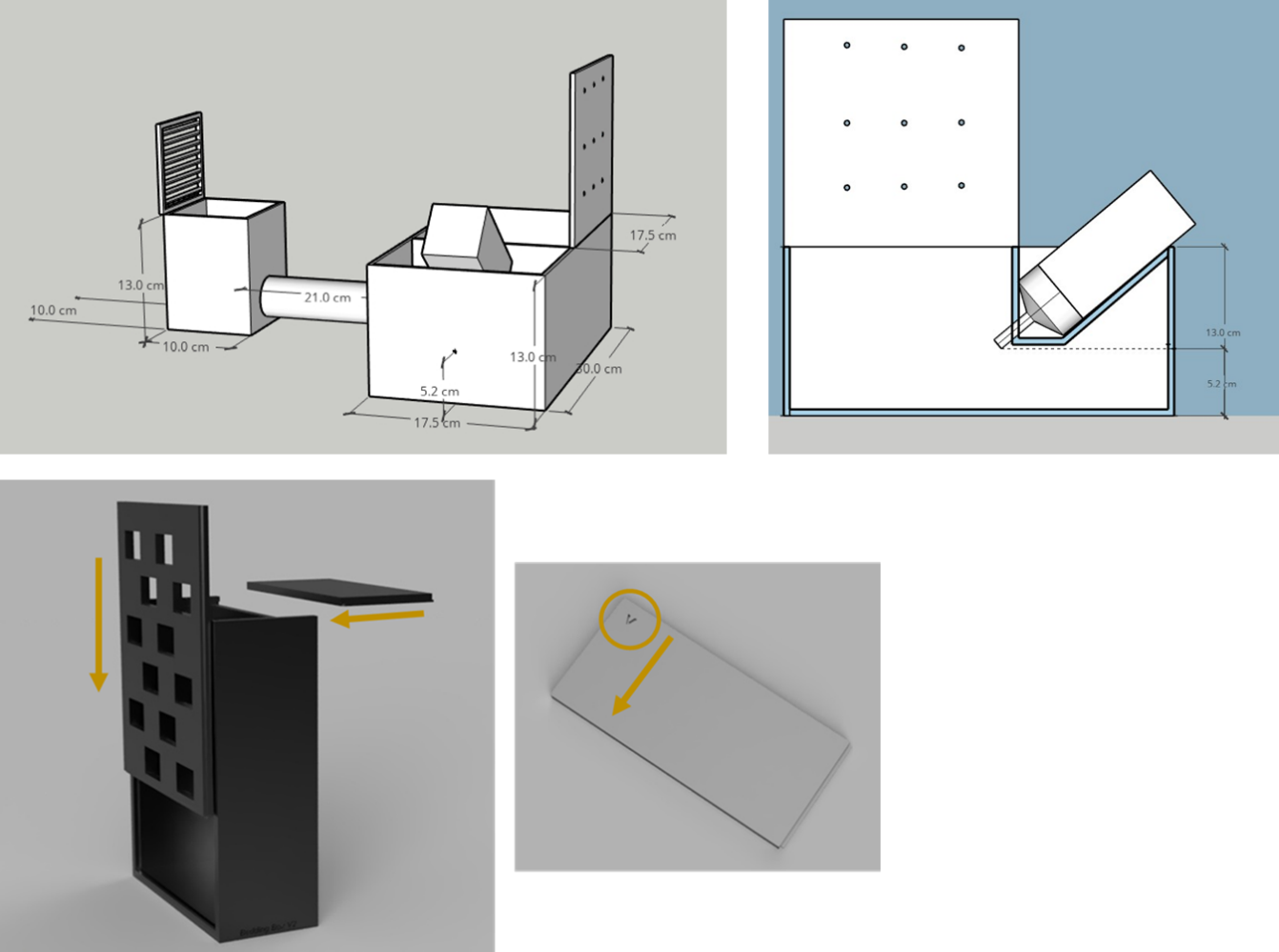


***S3. Dimensional drawings of arena and nesting material box components.*** *Dimensions of arena are internal. Lid is not hinged and is sat under its own weight and located using corner gussets. The use of solid Perspex creates a safe, enclosed environment. The forage area contains a barred floor, with woodchip underneath. It is covered with a detachable barred lid while in use. The nesting material box is fixed to the back of the forage area with magnet bar, which is external to the arena. The tube is detachable and mounted in a square bracket. The use of a barred floor in the forage area in combination with the more ‘open’ environment (use barred lid to allow air flow and clear Perspex) promotes shuttling of nesting material to the home area. All components are washable with warm water. Nesting material box components are 3D printable and are designed to slot together with minimal force. The nesting material box was 3D printed with black polylactic acid (PLA). While the individual aperture differed in size, the total available surface area for foraging remained the same between face plates by increasing the number of apertures available. Arrows indicate direction of construction. The lid has a small arrow engraved on the surface indicating the direction it should be slotted into the main body of the box. The faceplate should be slotted into the main body before the lid is slotted.*

**S4**

The dimensions of the strips should not exceed the dimensions of the smallest aperture, and should not fall through the aperture without being pulled. We highly recommend that the use of different nesting material is first piloted to assess practical suitability. Users should also be aware that previous work has shown that mice value different nesting materials differently (Bárdos, Boróka, István Nagy, Zsolt Gerencsér, and Vilmos Altbacker. 2022. "Nest Material Preference of Wild Mouse Species in Laboratory Housing" Applied Sciences 12, no. 11: 5750).

**S5**


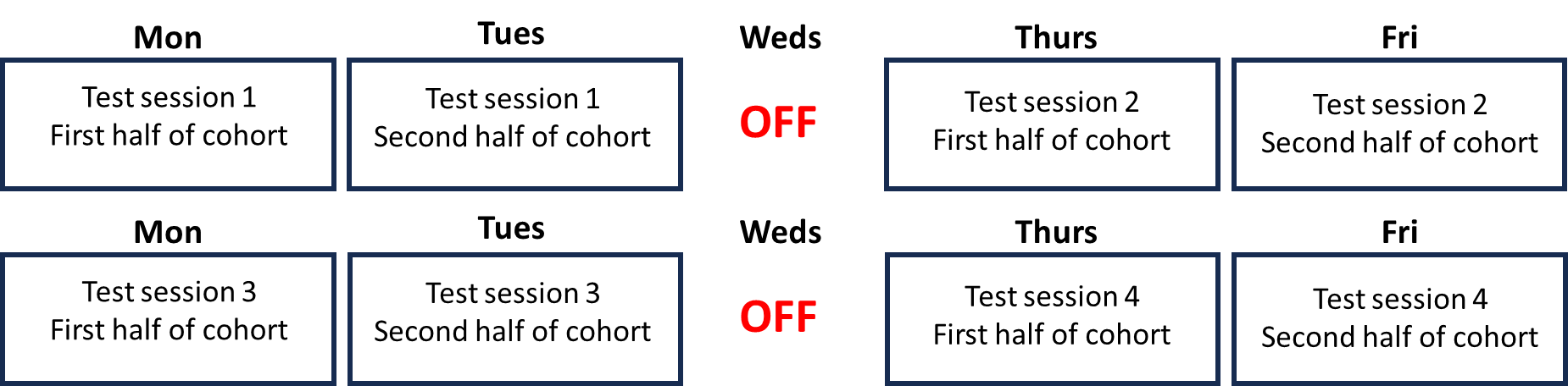


***S5. Acute pharmacological study test session design.*** *In a 3 dose plus vehicle design, testing takes place over 4 testing sessions (two weeks). Each test session is separated by at least 2 days to ensure drug washout between sessions.*

**S6**

**Drug vehicles**

| Drug | Dose range | Route of administration | Pre-treatment time | Vehicle |
| --- | --- | --- | --- | --- |
| Haloperidol | 0.01 – 0.1 mg/kg | i.p | 60 mins | 1% DMSO, 2% cremophore, 97% saline |
| Amphetamine | 0.1 – 0.3 mg/kg | i.p and oral | 15 mins | i.p-saline, oral-water + condensed milk (20%) |
| Methylphenidate | 1 – 10 mg/kg | oral | 15 mins | Water + condensed milk (20%) |

***Table 6.*** *Summary of acute drug studies. i.p – intraperitoneal, DMSO- dimethyl sulfoxide. Oral indicates voluntary ingestion of substance via syringe.*

**S7**

**Oral dosing training**

Briefly, mice were first habituated to a 20 % condensed milk solution (Nestlé Carnation) by putting ~ 50 µl on a surface in the home cage and letting them voluntarily consume it in their own time. The next day mice were given 300 µl condensed milk solution via a 1 ml syringe through the bars of the lid of the home cage. This was repeated until the mouse would approach the syringe and consume the solution in ~< 1 minute. On average this took 3 days.

**S8**

**General locomotor activity**

The system was built in-house by MGJ to monitor home cage activity in individually-housed mice, based on a system previously developed by (Brown, 2016). 4 activity sensors with built-in amplifiers (AMN 2,3,4 series Motion Sensor, Panasonic) were read using an Arduino Mega 2560 with an Arduino Mega daughter board (SchmartBoard, Mouser). Data was read onto an SD card as CSV file using an SD card adaptor (TFT LCD w/microSD Breakout, Adafruit) and exact time was outputted alongside the data using a RealTime clock (Clock & Timer Development Tools PCF8523 RTC for RPi, Adafruit). Each sensor was placed above the middle of the forage area, approximately 5 cm from the top of the cage. Movement was detected by the sensor every 100 msec. A percentage of movement within 10 secs was calculated and outputted in 10 second time bins. Area under the curve was calculated using time bin x activity count. Code to run the sensor system was adapted from (Brown, 2016) and can be found here <https://github.com/meganjackson13/Sensor-system-code>.

**S9**

| Experiment | Measure | Statistical exclusions in dataset |
| --- | --- | --- |
| Free nesting material | Total taken through | N = 1, std, outlier |
| Temp study | Total taken through | N = 1 pre-exclusion |
| Age: Habituation | Time spent in box(s) | N = 2 young, hab 1 (n = 1 missing value), n = 1 aged, hab 3 |
| Age: First session | Total taken through | No exclusions |
|  | % taken to main box | N = 1 young |
| Age: Effort curve | Total taken through | N = 1 pre-exclusion, n = 1 young, 1.5cm outlier |
|  | % taken to main box | N = 1 pre-exclusion, n = 1 young 0.75cm, n = 1 aged 1cm, outliers |
| Age: Big vs std | Total taken through | N = 1 pre-exclusion, n = 1 big, young, n = 1 big, aged, outliers |
|  | % taken to main box | N = 1 pre-exclusion, n = 1 std, young |
| CORT: First session | Total taken through | No exclusions |
|  | % taken through | N =1 (-) n = 1 (+) outliers |
| CORT: Effort curve | Total taken through | N = 1 pre-exclusion due to box error.  N = 1 0.75cm (-), n = 1 1cm (-), n = 1 1cm (+). |
|  | % taken to main box | N = 1 foraged 0g so % could not be calculated. |
| CORT: Big vs std | Total taken through | N = 1 big (-), n = 1 big (+), n = 1 std (+) outliers |
|  | % taken to main box | N = 1 big (-), n = 1 std (+) outliers |
| Male versus female | Total foraged | N = 1 male (1.5cm^2^), N = 1 female (0.75cm^2^) N = 1 female (1.5cm^2^) |
|  | Total taken through | N = 1 male (1.5cm^2^) |
|  | % taken to main box | N = 1 male (1.5cm^2^) |
| Haloperidol | Total taken through | N = 1 vehicle (box error) |
|  | Performance split | N = 1 0.03mg/kg outlier N = 1 0.1 mg/kg outlier |
|  | % taken to main box | N = 2 0.01mg/kg, n = 2 0.03 mg/kg, n = 1 0.1 mg/kg outliers. |
|  | Activity | N = 3 0.03mg/kg sensor error |
| Amphetamine i.p | Total taken through | N = 1 0.1 mg/kg outlier  N = 2 0.3 mg/kg outlier, missing value. |
|  | Performance split | N = 1 0.1 mg/kg. |
|  | % taken to main box | N = 1 excluded due to 3 outliers.  N = 1 0.1 mg/kg, n = 1 1.0mg/kg outliers. |
|  | Activity | N = 1 vehicle, n = 1 0.1 mg/kg, n= 2 (1 missing value) 0.3 mg/kg, n = 1 1.0mg/kg outliers |
| Amphetamine oral | Total taken through | N = 1 vehicle, outlier |
|  | % taken to main box | N = 1 vehicle, n = 1 0.1mg/kg, n = 1 0.3mg/kg outliers |
|  | Split | No exclusions |
| MPH oral | Total taken through | N = 2 (1 pre-excluded due to box error), 10 mg/kg, n = 1 vehicle, outliers |
|  | % taken to main box | N = 1 fully excluded due to multiple outlying points, n = 1 10mg/kg pre-exclusion as above. |
|  | Split | N = 1 1mg/kg, high, outlier, n = 1 pre-exclusion as above. |

***S9. Summary of data point exclusions/replacements per experiment.***

**S10**


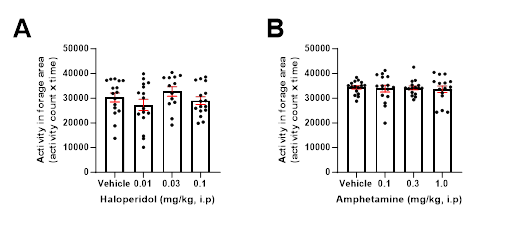


***S10. General locomotor activity in the forage area was unaffected by drug.*** *Activity was recorded in the forage area of the arena using a passive infrared sensor system.* *There was no effect of haloperidol or amphetamine (i.p) on general locomotor activity in the forage area (p > 0.05). Bars are mean ± SEM with data points overlaid.*

**S11**


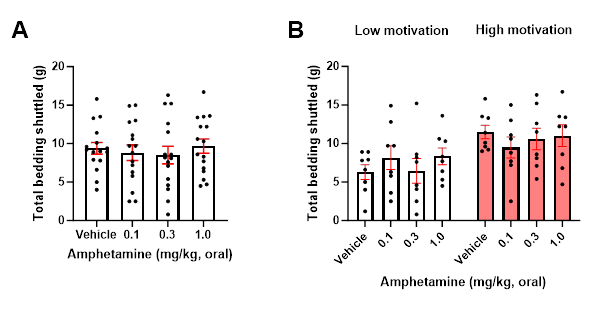


***S11. There was no effect of oral amphetamine on output measures.***

**S12**


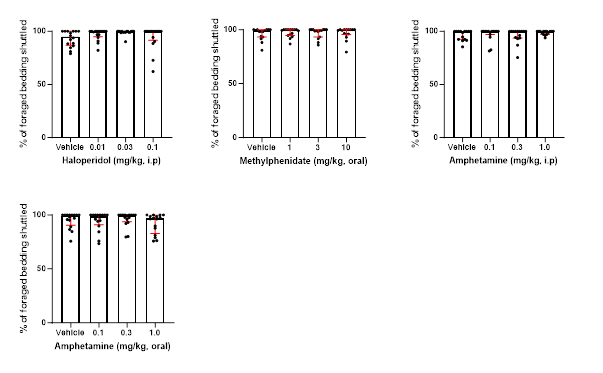


***S12. There was no effect of drug administration on % nesting material shuttled.***

**S13**

There was a main effect of aperture (F_(1.213,16.98)_ = 70.65, p < 0.0001) and sex (F_(1,14)_ = 54.22, p < 0.0001) on total nesting material foraged. There was no aperture*sex interaction. Post-hoc analysis revealed that females foraged less than males at the 0.75 cm^2^ aperture (p = 0.0027) and 1.5 cm^2^ aperture (p = 0.0076) but not the 1 cm^2^ aperture. Both males and females foraged more at the 1.5 cm^2^ aperture versus the 1 cm^2^ aperture (p = 0.0115 and p = 0.0043 respectively). Females foraged less at the 0.75 cm^2^ aperture versus the 1 cm^2^ aperture (p = 0.0043) (**S12.A**).

There was a main effect of aperture (F_(1.465, 20.51)_ = 35.69, p < 0.0001), sex (F_(1,14)_ = 54.22, p < 0.0001) and an aperture*sex interaction (F_(2,28)_ = 9.095, p = 0.0009) on total nesting material shuttled. Post-hoc analysis revealed females shuttled less nesting material than males at the 1.5 cm^2^ and 0.75 cm^2^ apertures (p = 0.0001) and trended towards a decrease at the 1 cm2 aperture (p = 0.0606). Males shuttled less nesting material at the 1 cm^2^ aperture versus the 1.5 cm^2^ (p = 0.0077) and females trended towards the same effect (p = 0.0603) (**S12.B**).

There was a main effect of aperture (F_(1.698, 23.77)_ = 8.31, p = 0.0028) and sex (F_(1,14)_ = 51.73, p < 0.0001). There was no sex*aperture interaction. Post hoc analysis revealed that males showed a greater % of foraged nesting material shuttled at all apertures compared to female mice (p ≤ 0.0042). There was no difference in % of foraged nesting material shuttled between apertures in either sex (**S12.C**).


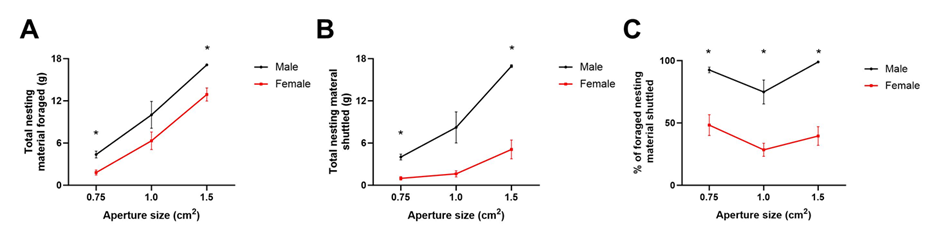


***S13.*** ***Male and female mice underwent the effort curve paradigm. A*** *Female mice forage less nesting material than males at the smallest and largest apertures.* ***B*** *Female mice shuttle less nesting material at the smallest and largest apertures.* ***C*** *Female mice show a reduced % of foraged nesting material shuttled at all apertures.*
